# Acute-onset Mania in a Patient with Non-small Cell Lung Cancer

**DOI:** 10.7759/cureus.5436

**Published:** 2019-08-20

**Authors:** Kate N Warren, Jhansi Katakam, Eduardo D Espiridion

**Affiliations:** 1 Medicine, West Virginia School of Osteopathic Medicine, Lewisburg, USA; 2 Psychiatry, Frederick Memorial Hospital, Frederick, USA

**Keywords:** mania, bipolar mania, brain tumor, steroid induced mania, brain metastasis

## Abstract

Mania is a mood disorder characteristic of certain psychiatric conditions and is exhibited by high energy, elevated mood, irritability, insomnia, and pressured speech. Though commonly attributed to bipolar and schizoaffective disorders, mania may be precipitated by other non-psychiatric conditions, including substance abuse, medications, metabolic disturbance, and organic brain pathology. Steroid-induced mania is not uncommon and may present with a number of psychiatric symptoms. Brain tumors presenting with predominantly psychiatric symptoms are a relatively uncommon cause of mania and may persist or recede with treatment. A case of mania in a cancer patient with brain metastasis and steroid use, with no prior history of mania, is discussed herein.

## Introduction

A manic episode presents as a distinct state of elevated mood with increased energy, which lasts for one week but may be present for a shorter duration if hospitalization is required. The following behavior may be present with mania: grandiosity, lack of sleep, increased speech, racing thoughts, distractibility, psychomotor agitation, and engagement in risky activities [1]. These characteristics, along with marked impairment and an inability of the behavior to be attributed to another psychological condition, medication, or medical illness constitutes the diagnosis of bipolar I disorder [1]. Mania is often associated with bipolar I disorder but may be precipitated by a number of other circumstances, including steroid use and organic brain pathology.

## Case presentation

**Figure 1:**
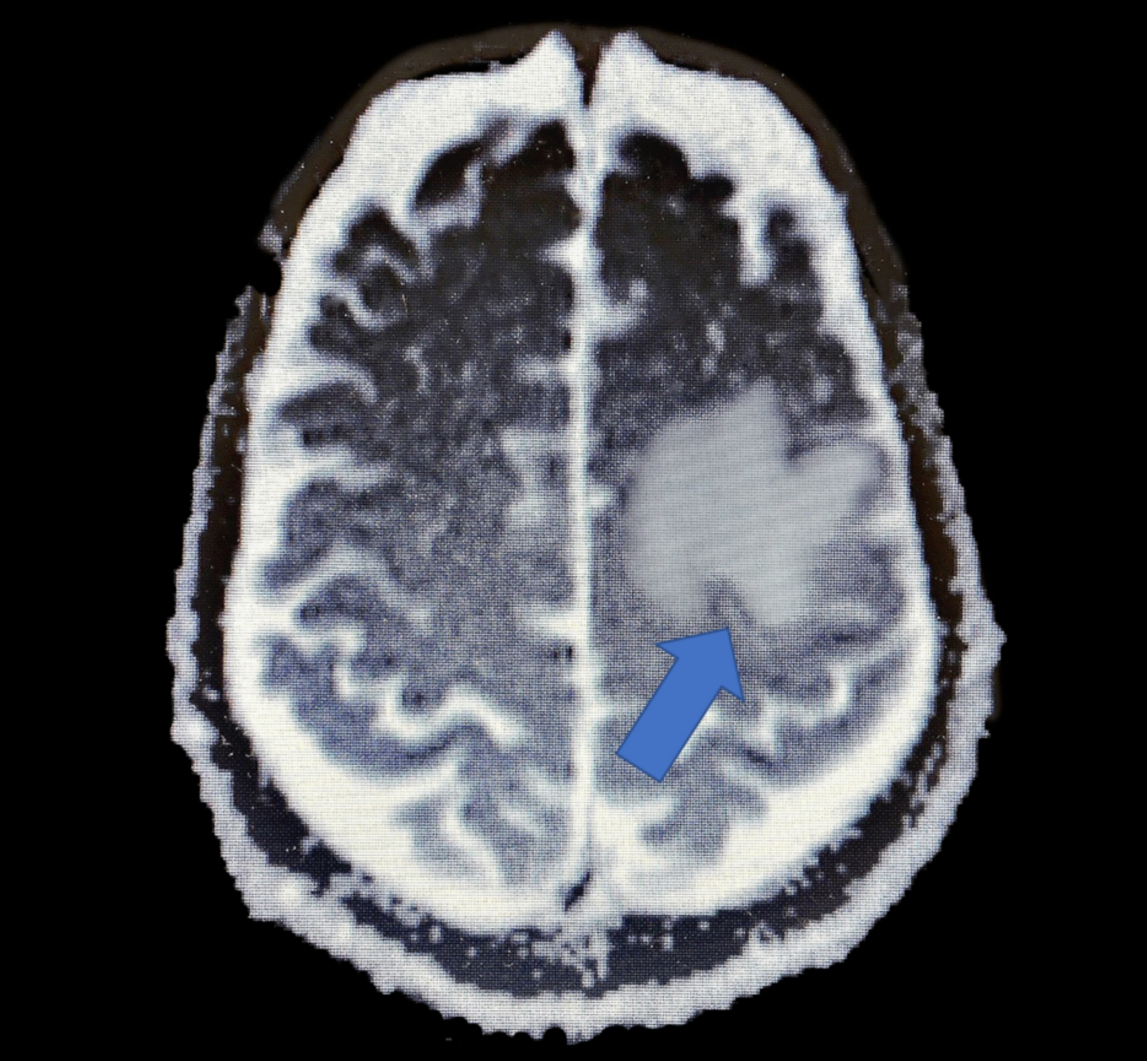
Pre-craniotomy MRI showing an enhancing mass with associated vasogenic edema in the posterior left frontal lobe consistent with brain metastasis.

A 65-year-old Caucasian male with metastatic stage III non-small cell lung cancer to the brain was brought to the hospital due to a sudden change of mental status. In September of 2017, the patient was diagnosed with programmed death-ligand 1 (PD-L1) positive adenocarcinoma of the lung and began a treatment regimen of carboplatin, paclitaxel, and durvalumab (IMFINZI). He completed his chemotherapy and stopped the durvalumab in January of 2019 due to gastrointestinal side effects. In June of 2019, the patient reported headaches to his oncologist and subsequent head computed tomography (CT) showed brain metastasis (Figure 1).

Later that month, the patient underwent craniotomy and biopsy, which was positive for a high-grade malignant tumor, consistent with metastatic adenocarcinoma of the lung. He was started on 4 mg of dexamethasone three times a day upon discharge from the hospital and was scheduled for CyberKnife (Accuray Incorporated, California, US) radiation therapy. One week after he arrived home, the patient experienced insomnia, excessive coffee consumption of multiple cups per hour, chain-smoking, and displayed increased hyperverbal behavior. This prompted his family to return to the hospital, stating that he was unmanageable.

The patient’s past medical history was significant for anxiety, depression, hypertension, hyperlipidemia, chronic obstructive pulmonary disease, coronary artery disease, coronary artery bypass graft, arrhythmia, chronic back pain, degenerative disc disease, depression, gastroesophageal reflux disease, gastrointestinal bleed, and peripheral vascular disease. He had been followed by an outpatient psychiatrist for anxiety and depression since 2012, for which he was prescribed 20 mg of escitalopram and 2 mg of clonazepam. In addition to dexamethasone and his previous psychiatric medication, the patient was taking lisinopril, albuterol, oxycodone, tiotropium, levetiracetam, atorvastatin, divalproex sodium, and hydromorphone. The patient had no prior history of manic or bipolar symptoms, no family history of mania, and no recent adjustments to his outpatient medications.

**Figure 2:**
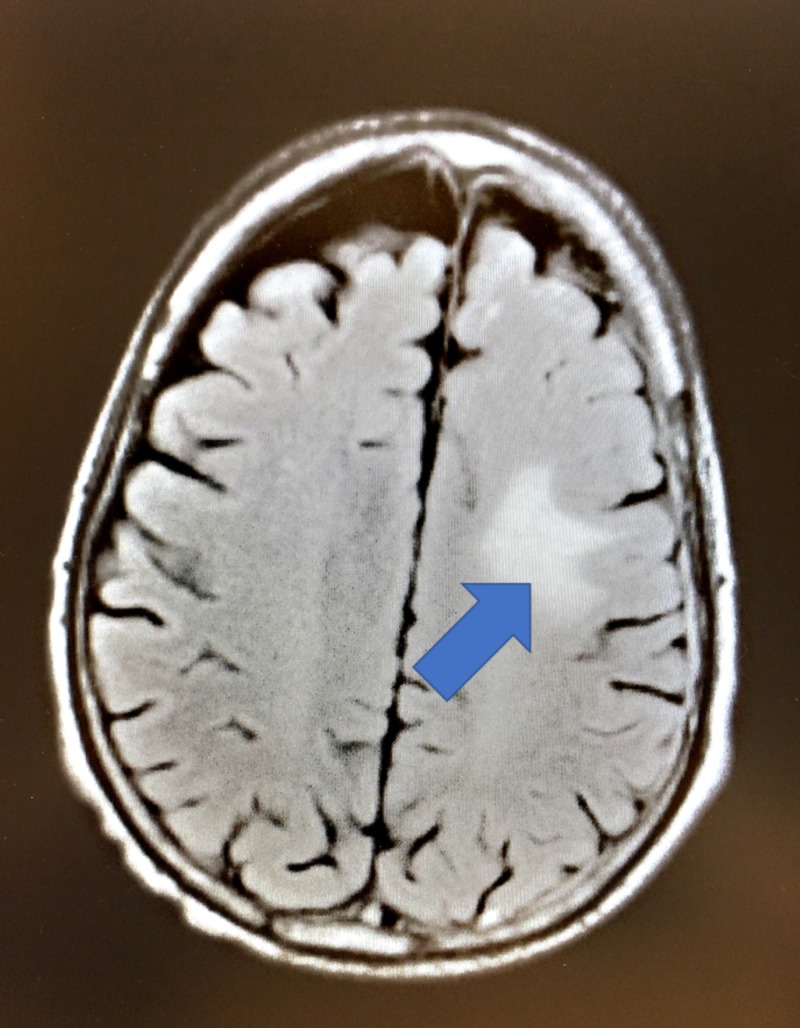
Post-craniotomy MRI showing a stable, small hemorrhage in the left frontoparietal region with persistent associated vasogenic edema. MRI: magnetic resonance imaging

On presentation to the emergency room, the patient appeared thin, cachectic, and agitated. His blood pressure was 150/84 mmHg, temperature was 97.8° F, pulse was 97 beats per minute, respiratory rate was 14 per minute, and pulse oximetry reading was 98% on room air. He was alert and oriented with no gross neurological deficits, though the patient was noted to be very agitated, hyperactive, and excessively verbal. Labs were drawn, which were all within normal limits except stable hemoglobin and hematocrit levels of 11.4 L and 34.0, respectively. Urine toxicology and alcohol screening were negative. Neurosurgery and psychiatry were consulted. Magnetic resonance imaging (MRI) of the brain was performed, which revealed a stable hemorrhage at the post-craniotomy site in the left parietal lobe, measuring 12 x 16 mm (Figure 2).

There was mild associated vasogenic edema and scattered foci of increased signal within the white matter, which was not significantly changed from his prior study. The patient was assumed to have steroid-induced mania, or mania secondary to brain metastasis. Neurosurgery decreased his dose of dexamethasone to 2 mg twice a day, and the patient was maintained on 750 mg of divalproex per day due to his manic symptoms.

Psychiatry noted the patient to have an illogical thought process with poor computation and poor short- and long-term memory, labile mood, and affect, fluent language, poor fund of knowledge, and lack of insight. The patient was discussed in the treatment team through the consultation-liaison psychiatry service and pharmacological management was recommended. They determined that the patient did not have medical decision-making capacity and prescribed 5 mg of olanzapine twice a day. The patient was admitted to the medical floor for observation.

During his hospital stay, the patient displayed increasingly agitated and labile behavior and was noted to be hyperverbal, perseverating, and constantly requiring redirection when questioned. He reported planning for eye surgeries, teeth extractions, spending money, and had plans to become a born-again Christian. He exhibited mood lability with alternating episodes of irritability and crying. He was noted to have racing thoughts and psychomotor agitation. He began insulting the hospital staff, refusing medication, and was found walking half-naked on the hospital floor, crying and asking the hospital staff to pray with him. He then requested the staff repeat after him that “daddy is coming home” and “you’re my second angel.”

After three days in the hospital, the patient was discharged to an inpatient psychiatric rehabilitation center with no noted improvement in his manic behavior.

## Discussion

The patient presented to the hospital with symptoms of mania, which is often attributed to an underlying psychiatric condition such as bipolar disorder. Bipolar disorder has a lifetime adult prevalence of 4%; most patients with the disorder have their first manic or depressive episode during adolescence or early adulthood, with a mean reported age of 18.2 years [2-3]. Bipolar may be categorized into early-onset, late-onset, and older-age bipolar disorder, as described by the International Society for Bipolar Disorder [4]. Early-onset is generally considered to be any patient who presents with an initial manic or hypomanic episode before the age of 50, late-onset is after the age of 50, and older-age onset is after the age of 60 [4]. Although most cases of bipolar disorder will present before the age of 50, up to 5%-10% will present with late-onset bipolar [4]. The age of onset of bipolar disorder may be due to a family history or genetic linkage in younger patients or occur after a diagnosis of a cerebrovascular incident or other brain pathology in older adults [4]. Though it has been suggested that there may be a link between cerebrovascular incidents and the onset of bipolar disorder in older adults, literature is currently lacking in this area.

The differential diagnosis for a patient with an acute onset of manic symptoms is broad and encompasses both psychiatric etiologies and organic causes, including metabolic, neurologic, and toxic disorders. A review of the patient’s past medical history, family and social history, and medications are crucial in contributing to the diagnosis. In every patient, but especially the elderly where new-onset bipolar I disorder is less common, organic etiologies must be excluded. A diagnosis of new-onset bipolar disorder was considered in this patient given his history of depression prior to his onset of manic symptoms. However, it should be noted that the patient’s anxiety and depression had been stable on maintenance escitalopram and clonazepam. While the diagnosis of lung cancer and brain metastasis might have exacerbated his anxiety and/or depression, worsening psychiatric symptoms were not noted in any of his oncology reports, and, importantly, the patient had never experienced a prior manic episode. These considerations along with the precipitation of manic symptoms after initiating steroids, and the lower likelihood of new-onset mania in this patient’s age group, made the diagnosis of new-onset bipolar disorder less likely.

Psychiatric symptoms may be the predominant symptomatology of organic brain pathologies, including brain tumors, infection, encephalitis, and stroke, but this is rare [5]. This patient had brain metastasis to the frontoparietal area and no other underlying central nervous system pathology that may have accounted for his manic symptoms. Although there is no proven correlation between the location of the lesion and development of psychiatric symptoms, mania appears most commonly with lesions in the right frontal lobe [5]. The treatment of mania secondary to brain tumors may involve the resection of the tumor, chemotherapy, or radiation [5]. Depending on the patient presentation, medications, including antipsychotics, may be necessary to control symptoms before treatment of the underlying process is initiated[5].Despite treatment, psychiatric symptoms may persist or even recur later in life. Currently, the literature is lacking on treatment guidelines for patients with persistent psychiatric symptoms secondary to brain tumors, and treatment is guided by an assessment of risks versus benefits, patient comorbidities, and potential pharmacologic interaction [5].

In this case, the patient’s manic symptoms began after being placed on oral dexamethasone, a long-acting, potent glucocorticoid steroid. The use of glucocorticoid therapy in medicine has been well-documented and is multifold; it is commonly used for inflammatory, neoplastic, as well as autoimmune conditions, including asthma, endocrinopathies, and in cancer regimens as an adjunct to chemotherapy [6-7]. Glucocorticoid-induced psychiatric symptoms are not uncommon and range from anxiety and depression, to mania, psychosis, and dementia. Manic and hypomanic symptoms are the most common [7]. Serious psychiatric symptoms occur in up to 6% of patients taking corticosteroids, usually within days to weeks of starting the medication [8]. Symptoms tend to be dose-dependent and decrease as the medication is tapered or discontinued [7]. Although the mechanism for steroid-induced mania is unclear, it has been theorized that corticosteroid use decreases the levels of corticotropin, norepinephrine, and beta-endorphin immunoreactivity[9]. Treatment of steroid-induced mania includes stopping the corticosteroids if feasible and starting medications such as mood stabilizers, including divalproex and lithium, and atypical antipsychotics such as quetiapine, carbamazepine, and olanzapine [7].

The patient had been taking divalproex sodium as seizure prophylaxis after his craniotomy but was maintained on this medication while in the hospital to treat his manic symptoms while being tapered off of steroids. Divalproex sodium (Depakote) is an enteric-coated compound made up of equal parts valproic acid and sodium valproate [10].Divalproex was first introduced into the United States in 1983 as an antiepileptic but was later Food and Drug Administration (FDA) approved for the treatment of acute mania in 1995 after a large clinical trial found it to be effective in this patient population [11].Although its mechanism of action in mania is poorly understood, it is hypothesized to potentiate the effects of gamma-aminobutyric acid (GABA), thereby inhibiting dopamine in the central nervous system [10].Prescriptions of divalproex peaked in the 1990s but have since declined due to the greater efficacy of atypical antipsychotics as well as certain side effects, including liver toxicity, pancreatitis, and gastrointestinal disturbance [11]. Divalproex sodium has been shown in numerous instances to be effective in the treatment and/or maintenance of steroid-induced mania [12-14].

## Conclusions

The patient with a history of metastatic brain cancer presented to the hospital status post craniotomy, recently started on dexamethasone, with a new onset of symptoms that met the Diagnostic and Statistical Manual of Mental Disorders’ criteria for a manic episode. This particular case was complicated by the fact that the patient had a number of comorbid conditions that could cause acute mania. His prior history of depression, recent steroid use, and history of brain metastasis could individually or in combination be causative or contributing factors for his acute onset of mania. Given the onset of symptoms within weeks of starting high dose steroids, it is reasonable to attribute his change of mental status to the steroids. They were appropriately lowered, and he was maintained on divalproex and olanzapine to stabilize his mood. Regardless of the cause, acute mania may be treated by a number of medications, including divalproex sodium, lithium, and atypical antipsychotics. Further treatment and maintenance of this patient will depend upon his response to treatment while in the inpatient psychiatric facility and will be determined by the supervising psychiatrist.
